# Возможности повышения приверженности к терапии пациентов с избыточной массой тела и ожирением в реальной клинической практике

**DOI:** 10.14341/probl13737

**Published:** 2026-03-07

**Authors:** Е. А. Трошина, Л. А. Суплотова, Ф. Х. Дзгоева, Е. В. Екушева, Е. С. Вьючнова, С. В. Оковитый, И. Г. Бакулин, Н. В. Бакулина, Т. Л. Каронова, Г. Р. Вагапова, Т. П. Бардымова, М. Р. Оразов, О. А. Радаева, З. Р. Гусова, Н. А. Мациевский, С. А. Сметанина, З. Г. Татаринцева, Ю. А. Катушкина

**Affiliations:** Национальный медицинский исследовательский центр эндокринологии им. акад. И.И. ДедоваРоссия; Endocrinology Research CentreRussian Federation; Тюменский государственный медицинский университетРоссия; Tyumen State Medical UniversityRussian Federation; Академия постдипломного образования ФГБУ «ФНКЦ ФМБА России»Россия; Academy of Postgraduate Education of the Federal State Budgetary Institution Federal Scientific and Clinical Center of the Federal Medical and Biological Agency of RussiaRussian Federation; Российский университет медициныРоссия; Russian University of MedicineRussian Federation; Санкт-Петербургский государственный химико-фармацевтический университет; Санкт-Петербургский государственный университетРоссия; Saint Petersburg State Chemical Pharmaceutical University; Saint Petersburg State UniversityRussian Federation; Северо-Западный государственный медицинский университет им. И.И. МечниковаРоссия; North-Western State Medical University named after I.I. MechnikovRussian Federation; Научный медицинский исследовательский центр им. В.А. Алмазова в РоссииРоссия; V.A. Almazov National Medical Research Center of EndocrinologyRussian Federation; Казанский государственный медицинский университетРоссия; Kazan State Medical UniversityRussian Federation; Иркутская государственная медицинская академия последипломного образованияРоссия; Irkutsk State Medical Academy of Postgraduate EducationRussian Federation; Российский университет дружбы народов им. Патриса ЛумумбыРоссия; Patrice Lumumba Peoples’ Friendship University of RussiaRussian Federation; Национальный исследовательский Мордовский государственный университет имени Н.П. ОгарёваРоссия; Federal State Budgetary Educational Institution of Higher Education, National Research Ogarev Mordovia State UniversityRussian Federation; Ростовский государственный медицинский университетРоссия; Rostov State Medical UniversityRussian Federation; Кубанский государственный медицинский университетРоссия; Kuban State Medical UniversityRussian Federation

**Keywords:** ожирение, семаглутид, тирзепатид, obesity, semaglutide, tirzepatide

## Abstract

26 сентября 2025 года во Владикавказе прошло заседание экспертной междисциплинарной рабочей группы, посвященное обсуждению вопросов повышения приверженности к терапии современными инкретинами пациентов с избыточной массой тела и ожирением в реальной клинической практике.

## Abstract (en)

On September 26, 2025, a meeting of an expert multidisciplinary working group was held in Vladikavkaz, dedicated to discussing ways to improve therapy adherence to modern incretin-based treatments in patients with overweight and obesity in real-world clinical practice.

## Введение

В последние годы ожирение и метаболические нарушения приобрели характер общемировой неинфекционной эпидемии, оказывая существенное влияние на частоту инвалидизации, рост сердечно-сосудистых и онкологических заболеваний, снижение трудоспособности и качества жизни населения. Согласно последним прогнозам, к 2035 г. до 75% взрослого населения в мире могут иметь избыточную массу тела или ожирение — это приведет к беспрецедентным нагрузкам на системы здравоохранения и требует глубокого пересмотра стратегий профилактики и лечения [1].

Современные стандарты ведения пациентов с ожирением ориентированы на интеграцию самых разных дисциплин: терапии, эндокринологии, кардиологии, гастроэнтерологии, психологии, диетологии, реабилитации и др., что обусловлено сложностью патогенеза, многочисленностью клинических фенотипов и высокой частотой сопутствующих заболеваний. С появлением новых глюкагоноподобных препаратов — агонистов глюкагоноподобного пептида 1 (арГПП-1) семаглутида в дозировке 2,4 мг (Велгия ЭКО) и двойного агониста глюкагоноподобного пептида и глюкозозависимого инсулинотропного полипептида (арГПП-1+ГИП) тирзепатида (Тирзетта), — продемонстрировавших не только выраженную эффективность по снижению массы тела, но и убедительные метаболические и кардиопротективные эффекты. Однако практика показывает, что, несмотря на наличие международных и национальных клинических рекомендаций, значительный пласт реальных вопросов, с которыми врач ежедневно встречается при ведении пациентов с ожирением, остается за пределами алгоритмов и стандартов [2][3][4].

## Остаются нерешенными многие вопросы

Эти и многие другие вопросы формируют повестку современного научного поиска и требуют выработки гибких, прозрачных и обоснованных решений, способных лечь в основу новых протоколов клинической практики.

Представленная далее резолюция экспертного совета основывается на стратегиях персонализированной терапии, оценке рисков, алгоритмах преодоления типовых и сложных клинических ситуаций в лечении пациентов с ожирением и отражает подходы, выработанные в междисциплинарном диалоге.

## Портрет пациента для назначения селективных и комбинированных аГПП-1 с позиции доказательной медицины

Выбор между семаглутидом и тирзепатидом и решение о начале терапии в целом — это не просто сравнение процентов снижения веса, а стратифицированный и персонализированный подход, основанный на комплексной оценке пациента. Ключевая идея: сегодня мы лечим не просто ожирение, а метаболическое заболевание «ожирение» с целью профилактики и снижения рисков его многочисленных и тяжелых осложнений, а также увеличения продолжительности жизни.

Эксперты предложили определенную иерархию критериев для назначения терапии (от наиболее к наименее значимым):

## Коморбидная патология — определяющий критерий

Наличие сопутствующих заболеваний — это не просто дополнительный фактор, а основное показание для рассмотрения терапии новейшими инкретинами, именно здесь проявляется их максимальный плейотропный эффект.

Приоритет №1(Доказанная эффективность по жестким конечным точкам).

Приоритет №2(Высокий риск прогрессирования).

## Индекс массы тела (ИМТ) — количественный пороговый критерий

ИМТ задает «точку входа» в терапию, но его интерпретация достаточно гибкая.

## Предыдущий опыт терапии

Пациент, который прошел через многократные неудачные попытки снизить вес с помощью диет, изменения образа жизни и, возможно, других фармакологических средств с меньшей эффективностью, — идеальный кандидат. Это демонстрирует не «слабоволие» пациента, а биологическую природу заболевания, требующую более мощного медикаментозного вмешательства.

## Возраст — критерий модификации тактики, а не противопоказания

Пациенты 18–65 лет: стандартный подход к титрации и целям.

Пациенты >65 лет (особенно >70): требуется особо осторожный подход:

В реальной клинической практике врач исходит из комплексной картины: анализирует не только абсолютное значение массы тела и имеющиеся осложнения, но и тип распределения жировой ткани (висцеральное ожирение), выраженность коморбидности, динамику веса, общее метаболическое состояние и индивидуальные риски. Для каждого пациента определяются целевые параметры (% или абсолютные показатели снижения массы тела, показатели компенсации коморбидности), стратегии сопровождения (физическая активность, коррекция питания, нутритивная и антисаркопеническая поддержка у пожилых и мужчин).

Итак, современный профиль кандидата на терапию семаглутидом или тирзепатидом — это взрослый человек с ожирением (или ИМТ≥27 кг/м² при коморбидных состояний), испытывающий выраженные трудности контроля веса или метаболических показателей, невзирая на применявшиеся ранее методы, с учетом возраста, пола, структуры массы тела, персонализированного риска и медицинских целей терапии. Такой комплексный персонализированный подход повышает как безопасность, так и клиническую эффективность современных препаратов для снижения массы тела и коррекции метаболических нарушений.

## Титрация дозы арГПП-1 и определение целей терапии: персонализированный подход

Этот вопрос является центральным в практическом применении инкретиновой терапии. Эксперты единогласно подчеркивали, что подход должен быть гибким и индивидуальным, отходящим от жестких протоколов в сторону стратегического планирования и управления коморбидными состояниями для улучшения качества жизни пациентов.

Многокомпонентные цели терапии: от килограммов к комплексному метаболическому улучшению.

Целевые показатели при лечении арГПП-1 следует рассматривать как иерархическую систему, где снижение массы тела является фундаментом для достижения более значимых клинических результатов.

## Цели, связанные с массой тела

## Метаболические и кардиоваскулярные цели

## Цели, связанные с качеством жизни

## Стратегия титрации доз: дифференцированный подход к семаглутиду и тирзепатиду

Современная тактика титрации арГПП-1 требует дифференцированного подхода в зависимости от конкретного препарата. Анализ данных клинических исследований и реальной практики позволяет сформулировать четкие рекомендации.

## Семаглутид: обязательное достижение целевой дозы 2,4 мг

Обоснование: клиническая эффективность семаглутида в лечении ожирения была доказана в исследованиях STEP именно на дозе 2,4 мг. Эта доза является целевой и зарегистрированной для данной патологии.

Тактика: терапия должна быть инициирована с дозы 0,25 мг с последующим с увеличением дозировки раз в месяц по стандартной схеме (0,5 мг → 1,0 мг → 1,7 мг → 2,4 мг) до достижения полной терапевтической дозы. Прерывание титрации на промежуточных дозах (1,0 мг, 1,7 мг) не рекомендуется, так как эффективность этих доз для снижения веса уступает целевой дозе 2,4 мг.

Исключения: временное замедление титрации или возврат на предыдущую дозу допустимы только при развитии непереносимых побочных эффектов с последующей повторной попыткой достижения дозы 2,4 мг после их купирования [12].

## Тирзепатид: три гибких режима дозирования (5 мг, 10 мг, 15 мг)

Обоснование: в отличие от семаглутида, тирзепатид демонстрирует дозозависимую эффективность в широком диапазоне доз (5 мг, 10 мг, 15 мг), что подтверждено исследованиями SURMOUNT. Это предоставляет врачу возможность выбора конечной дозы в зависимости от индивидуальных целей терапии и переносимости [10].

Три режима дозирования и критерии выбора:

Дифференцированный подход основан на принципах доказательной медицины и данных реальной клинической практики — для семаглутида он достигается только на дозе 2,4 мг, в то время как для тирзепатида существует несколько терапевтических режимов, что расширяет возможности персонализации лечения и управления побочными эффектами.

## Алгоритм отмены арГПП-1: стратегия предотвращения рецидива

Отмена инкретиновой терапии является критическим этапом, неправильное проведение которого сводит на нет все достигнутые результаты. Консенсус экспертов основан на понимании ожирения как хронического рецидивирующего заболевания, требующего долгосрочного управления.

Патофизиологическое обоснование: возможные риски одномоментной отмены.

Ожирение — это состояние, при котором организм устанавливает новую, более высокую «метаболическую точку отсчета». арГПП-1, воздействуя на центры голода и насыщения в гипоталамусе, а также замедляя опорожнение желудка, «перепрограммирует» эту точку.

Эффект рикошета: при резкой отмене происходит следующее:

Клиническое следствие резкой отмены: набор веса, причем часто до исходного уровня, так как организм стремится восстановить утраченные жировые запасы.

В качестве вариантов поддерживающей терапии при достижении целей можно рассмотреть алгоритм обратной титрации или удлинение промежутков между инъекциями:

Схема для препаратов с еженедельным введением (семаглутид, тирзепатид)

Этап 1: переход с инъекций 1 раз в 7 дней на введение 1 раз в 9–10 дней. Пациент находится в этом режиме в течение 1–2 месяцев.

Этап 2: при стабильном весе — удлинение интервала до 1 инъекции в 14 дней. Еще 1–2 месяца наблюдения.

Этап 3 (при успехе): попытка перехода на введение 1 раз в 3 недели с последующей полной отменой.

Преимуществами режима обратной титрации являются минимизация психологического дискомфорта для пациента, который продолжает получать терапию, возможность объективно оценить, как организм реагирует на снижение лекарственной поддержки, и при наборе веса можно легко вернуться к более короткому интервалу.

Важно понимать, что отмена терапии — не цель, а взвешенное клиническое решение, которое имеет четкие показания.

Показания для начала обратной титрации:

Показаниями к немедленному возврату к предыдущему этапу терапии являются:

Алгоритм отмены инкретинов должен быть таким же продуманным и индивидуальным, как и алгоритм их назначения. Поэтапное, контролируемое снижение лекарственной нагрузки, в первую очередь за счет удлинения интервалов между инъекциями, позволяет минимизировать риск рецидива и закрепить достигнутый успех на долгосрочную перспективу.

## Программа долгосрочного ведения пациентов после достижения целевых показателей снижения массы тела: стратегия поддержания результата

Достижение целевых показателей снижения массы тела при терапии инкретинами знаменует переход к критически важному этапу — долгосрочному поддержанию результата. Современная концепция лечения ожирения как хронического рецидивирующего заболевания требует разработки комплексной программы пожизненного ведения, направленной на предотвращение восстановления исходной массы тела [1].

Организационная модель динамического наблюдения — мультидисциплинарный подход.

Эффективное ведение пациента на этапе поддержания массы тела требует интеграции усилий различных специалистов:

Интенсивность мониторинга должна поэтапно снижаться при сохранении регулярности наблюдений (табл. 1).

**Table:** Таблица 1. План динамического наблюдения после достижения целевой массы тела

Период наблюдения	Частота визитов	Основные параметры контроля
1–3 месяца(фаза активной стабилизации)	Каждые 2 недели	Динамика массы тела и окружности талии, коррекция пищевого поведения
4–6 месяцев(фаза закрепления)	1 раз в месяц	Стабильность антропометрических показателей, оценка физической активности
После 6 месяцев(фаза пожизненного поддержания)	1 раз в 3 месяца	Комплексная оценка метаболических параметров, коррекция программы

Основу нутритивной поддержки составляют средиземноморская диета или DASH-диета, адаптированная под индивидуальные потребности; адекватное потребление белка (1,2–1,5 г/кг целевой массы тела) для профилактики саркопении; дробный режим питания (3 основных приема пищи + 1–2 перекуса).

Программа физических нагрузок включает аэробные нагрузки: 150–300 минут в неделю умеренной интенсивности; силовые тренировки: не менее 2 раз в неделю и использование фитнес-трекеров для объективного мониторинга активности.

## Механизмы контроля и оценки эффективности

Для объективной оценки соблюдения рекомендаций применяются:

## Распределение ответственности в системе «врач-пациент»

Эффективное долгосрочное поддержание достигнутых результатов снижения массы тела требует четкого распределения ответственности между медицинским работником и пациентом. Формирование партнерской модели взаимоотношений является критически важным условием успешной профилактики рецидива заболевания.

Ответственность врача включает:

Ответственность пациента включает:

Разработанная программа долгосрочного ведения пациентов после отмены арГПП-1 представляет собой комплексную систему, основанную на принципах мультидисциплинарного подхода, поэтапного динамического наблюдения и четкого распределения ответственности между врачом и пациентом. Критически важным элементом является готовность к немедленной коррекции плана при первых признаках рецидива заболевания.

## Критерии и алгоритм повторного назначения арГПП-1 при рецидиве ожирения

Ожирение является хроническим рецидивирующим заболеванием, что обусловливает высокую вероятность восстановления массы тела после прекращения терапии. Согласно данным, представленным в ходе экспертного обсуждения, до 65% пациентов прекращают терапию арГПП-1 в течение 2 лет, при этом только половина из них возобновляет лечение. Разработка четких критериев и алгоритмов повторного назначения терапии представляет собой важнейший компонент долгосрочного управления заболеванием.

## Критерии для повторного назначения терапии

Антропометрические критерии

Метаболические критерии

1) повышение уровня HbA1c ≥0,5% от достигнутого минимума;

2) появление нарушенной толерантности к глюкозе.

1) повышение уровня триглицеридов ≥1,7 ммоль/л;

2) снижение Х-ЛПВП <1,0 ммоль/л для мужчин, <1,2 ммоль/л для женщин.

Клинические критерии

Четкие критерии и стандартизированный алгоритм повторного назначения арГПП-1 при рецидиве ожирения позволяют оптимизировать долгосрочное ведение пациентов (табл. 2). Своевременное возобновление терапии при соблюдении установленных показаний способствует поддержанию достигнутых результатов и профилактике прогрессирования коморбидных состояний. Согласно консенсусу экспертов, ключевым моментом является готовность к быстрому возобновлению терапии при появлении первых признаков обратного набора веса (рецидива).

**Table:** Таблица 2. Алгоритм повторного назначения арГПП-1 при рецидиве ожирения

Этап	Мероприятия	Критерии перехода к следующему этапу
1. Оценка показаний	- Измерение массы тела, ИМТ, ОТ- Оценка метаболических параметров- Анализ причин рецидива	Выявление ≥1 критериев для повторного назначения
2. Выбор тактики	- Определение целевых показателей - Оценка предыдущего опыта терапии- Выбор препарата и стартовой дозы- Коррекция немедикаментозной терапии	Разработка индивидуального плана терапии
3. Инициация терапии	- Назначение препарата в стартовой дозе- Обучение технике инъекций- Информирование о возможных ПЯ	Отсутствие противопоказаний
4. Титрация	- Постепенное увеличение дозы согласно схеме- Мониторинг переносимости- Коррекция сопутствующей терапии	Достижение целевой терапевтической дозы

## Алгоритм отмены арГПП-1 при планировании беременности и ведение случаев наступления беременности на фоне терапии

Вопрос применения инкретинов при планировании и наступлении беременности остается сложным и достаточно актуальным. Согласно действующим инструкциям, препараты этой группы противопоказаны при беременности, однако реальная клиническая практика требует четкого алгоритма действий на этапах подготовки к зачатию как со стороны женщины, так и мужчины.

Алгоритм отмены терапии при планировании беременности: в соответствии с официальной инструкцией, препараты необходимо отменять за 2 месяца до потенциального зачатия как у женщин, так и у мужчин. Двухмесячный период отмены основан на фармакокинетических характеристиках препаратов и обеспечивает полную элиминацию лекарственного средства из организма.

Важно отметить, что согласно результатам систематического обзора, представленного в ходе экспертного обсуждения, не выявлено статистически значимого увеличения риска пороков развития при случайном приеме аГПП-1 на ранних сроках беременности, риск самопроизвольных выкидышей и преждевременных родов сопоставим с общей популяцией.

Важно помнить, что во время применения препаратов агПП-1 рекомендуется использовать методы контрацепции.

Рекомендуется строгое соблюдение официальных рекомендаций по отмене препаратов за 2 месяца до планируемого зачатия. Случайное применение препаратов на ранних сроках беременности не должно рассматриваться как показание к ее прерыванию.

## Ожидаемые и реальные эффекты терапии арГПП-1 на сердечно-сосудистую систему

Согласно данным, представленным в ходе экспертного обсуждения, влияние аГПП-1 на сердечно-сосудистую систему имеют двойной характер, сочетая доказанные кардиопротективные эффекты с отдельными клинически значимыми функциональными изменениями [1]. Многоцентровые рандомизированные исследования продемонстрировали значимое влияние терапии аГПП-1 на кардиоваскулярные исходы:

Основными механизмами кардиопротекции являются прямое влияние на кардиомиоциты через рецепторы ГПП-1, улучшение эндотелиальной функции, снижение системного воспаления, нормализация уровня липидов крови и гликемии.

В реальной клинической практике участники экспертного совета отметили, что пациенты могут испытывать учащение сердечного ритма на 2–4 удара в минуту в среднем, что не требует отмены терапии.

Таком образом, доказано, что терапия аГПП-1 демонстрирует значимые кардиопротективные эффекты, подтвержденные данными крупных исследований. Однако в реальной клинической практике необходимо учитывать возможность развития тахикардии у ряда пациентов.

## Алгоритм назначения урсодезоксихолевой кислоты (УДХК) при терапии арГПП-1: тактика и сроки

Применение агонистов рецепторов глюкагоноподобного пептида-1 (аГПП-1) ассоциировано с повышенным риском развития желчнокаменной болезни (ЖКБ) и билиарных осложнений, что обусловлено быстрым снижением массы тела и изменением физико-химических свойств желчи. Препараты УДХК рассматриваются как ключевой элемент профилактики и коррекции данных осложнений.

Алгоритм назначения УДХК основан на стратификации пациентов по риску развития билиарных осложнений (табл. 3).

**Table:** Таблица 3. Факторы риска развития желчнокаменной болезни при терапии аГПП-1

Категория риска	Факторы риска	Тактика назначения УДХК
Высокий риск	- Наличие сладжа/микролитиаза- ЖКБ в анамнезе- Быстрое снижение веса (>1,5 кг/неделю)- Женский пол, возраст >40 лет - НАЖБП	Обязательное назначение со старта терапии
Умеренный риск	- Ожирение II–III степени- Сахарный диабет 2 типа- Нарушения липидного обмена	Рассмотреть назначение со старта терапии
Низкий риск	Отсутствие перечисленных факторов риска	Назначение при появлении факторов риска

Согласно консенсусу экспертов, оптимальным считается начало терапии УДХК одновременно со стартом терапии аГПП-1 у пациентов группы высокого риска. Обязательно назначение УДХК при выявлении нарушений моторики желчного пузыря, сладжа или микролитиаза по данным УЗИ. Также необходимо рассмотреть назначение УДХК при темпе снижения веса >1,5 кг/неделю. Пациентам, перенесшим холецистэктомию, назначение УДХК показано для профилактики холелитиаза в желчных протоках и развития билиарного панкреатита

Режим дозирования и длительность терапии УДХК: оптимальный диапазон доз составляет 13–15 мг/кг/сутки. При плохой переносимости возможно снижение до 10 мг/кг/сутки. Длительность приема — на весь период активного снижения массы тела и период применения аГПП-1. Решение об отмене приема УДХК принимается индивидуально при стабилизации веса и отсутствии факторов риска.

Эксперты в ходе дискуссии отметили, что УДХК потенцирует эффекты арГПП-1 в отношении улучшения метаболических параметров, совместное применение позволяет повысить приверженность терапии ожирения за счет снижения побочных эффектов и улучшение переносимости лечения. Кроме того, УДХК может быть эффективной базисной терапией при наличии у пациентов НАЖБП, учитывая ее влияние на звенья патогенеза НАЖБП (аутофагия, оксидативный стресс и др.).

## Совместное применение прокинетиков и арГПП-1: стратегия профилактики и купирования диспепсических явлений

Диспепсические явления, включая тошноту, рвоту и гастропарез, являются наиболее частыми побочными эффектами терапии инкретинами. Согласно данным экспертного совета, эти эффекты служат основной причиной снижения приверженности терапии и преждевременного ее прекращения. Применение прокинетиков, в частности итоприда, представляет собой эффективный метод управления данными осложнениями.

Обоснование совместного применения (табл. 4):

Режимы дозирования итоприда:

Длительность терапии:

**Table:** Таблица 4. Показания к назначению прокинетиков при терапии аГПП-1

Клиническая ситуация	Тактика назначения	Уровень доказательности
Развитие умеренных и тяжелых диспепсических явлений	Назначение при появлении симптомов (по требованию)	Консенсус экспертов, данные клинических исследований
Гастропарез на фоне терапии	Обязательное назначение	Клинические рекомендации

Стоит обратить внимание, что выбор момента назначения должен определяться индивидуальными особенностями пациента и использоваться в качестве симптоматической терапии для купирования возникших диспепсических расстройств.

## Заключение и выводы

Семаглутид и тирзепатид являются высокоэффективными препаратами для лечения ожирения и управления метаболическим здоровьем. Выбор между ними должен быть персонифицированным.

## ДОПОЛНИТЕЛЬНАЯ ИНФОРМАЦИЯ

Источники финансирования. Работа выполнена по инициативе авторов без привлечения финансирования.

Конфликт интересов. Авторы декларируют отсутствие явных и потенциальных конфликтов интересов, связанных с содержанием настоящей статьи

Участие авторов. Все авторы одобрили финальную версию статьи перед публикацией, выразили согласие нести ответственность за все аспекты работы, подразумевающую надлежащее изучение и решение вопросов, связанных с точностью или добросовестностью любой части работы.

## References

[cit1] World Obesity Atlas 2025, https://www.worldobesity.org

[cit2] Nadolsky Karl, Garvey W. Timothy, Agarwal Monica, Bonnecaze Alex, Burguera Bartolome, Chaplin Michelle DeGeeter, Griebeler Marcio L., Harris Samantha R., Schellinger Jeffrey N., Simonetti Juliana, Srinath Reshmi, Yumuk Volkan (2025). American Association of Clinical Endocrinology Consensus Statement: Algorithm for the Evaluation and Treatment of Adults with Obesity/Adiposity-Based Chronic Disease – 2025 Update. Endocrine Practice.

[cit3] Lincoff A. Michael, Brown-Frandsen Kirstine, Colhoun Helen M., Deanfield John, Emerson Scott S., Esbjerg Sille, Hardt-Lindberg Søren, Hovingh G. Kees, Kahn Steven E., Kushner Robert F., Lingvay Ildiko, Oral Tugce K., Michelsen Marie M., Plutzky Jorge, Tornøe Christoffer W., Ryan Donna H. (2023). Semaglutide and Cardiovascular Outcomes in Obesity without Diabetes. New England Journal of Medicine.

[cit4] Lincoff A. Michael, Brown-Frandsen Kirstine, Colhoun Helen M., Deanfield John, Emerson Scott S., Esbjerg Sille, Hardt-Lindberg Søren, Hovingh G. Kees, Kahn Steven E., Kushner Robert F., Lingvay Ildiko, Oral Tugce K., Michelsen Marie M., Plutzky Jorge, Tornøe Christoffer W., Ryan Donna H. (2023). Semaglutide and Cardiovascular Outcomes in Obesity without Diabetes. New England Journal of Medicine.

[cit5] Packer Milton, Zile Michael R., Kramer Christopher M., Baum Seth J., Litwin Sheldon E., Menon Venu, Ge Junbo, Weerakkody Govinda J., Ou Yang, Bunck Mathijs C., Hurt Karla C., Murakami Masahiro, Borlaug Barry A. (2024). Tirzepatide for Heart Failure with Preserved Ejection Fraction and Obesity. New England Journal of Medicine.

[cit6] Kosiborod Mikhail N., Abildstrøm Steen Z., Borlaug Barry A., Butler Javed, Rasmussen Søren, Davies Melanie, Hovingh G. Kees, Kitzman Dalane W., Lindegaard Marie L., Møller Daniél V., Shah Sanjiv J., Treppendahl Marianne B., Verma Subodh, Abhayaratna Walter, Ahmed Fozia Z., Chopra Vijay, Ezekowitz Justin, Fu Michael, Ito Hiroshi, Lelonek Małgorzata, Melenovsky Vojtech, Merkely Bela, Núñez Julio, Perna Eduardo, Schou Morten, Senni Michele, Sharma Kavita, Van der Meer Peter, von Lewinski Dirk, Wolf Dennis, Petrie Mark C (2023). Semaglutide in Patients with Heart Failure with Preserved Ejection Fraction and Obesity. New England Journal of Medicine.

[cit7] Loomba Rohit, Hartman Mark L., Lawitz Eric J., Vuppalanchi Raj, Boursier Jérôme, Bugianesi Elisabetta, Yoneda Masato, Behling Cynthia, Cummings Oscar W., Tang Yuanyuan, Brouwers Bram, Robins Deborah A., Nikooie Amir, Bunck Mathijs C., Haupt Axel, Sanyal Arun J. (2024). Tirzepatide for Metabolic Dysfunction–Associated Steatohepatitis with Liver Fibrosis. New England Journal of Medicine.

[cit8] Phase 3 ESSENCE Trial: Semaglutide in Metabolic DysfunctionAssociated Steatohepatitis. Gastroenterol Hepatol (N Y). 2024;20(12 Suppl 11):6-7 PMC11784563 39896971

[cit9] McGowan Barbara M, Bruun Jens M, Capehorn Matt, Pedersen Sue D, Pietiläinen Kirsi H, Muniraju Hanna Angelene Kudiyanur, Quiroga Maria, Varbo Anette, Lau David C W (2024). Efficacy and safety of once-weekly semaglutide 2·4 mg versus placebo in people with obesity and prediabetes (STEP 10): a randomised, double-blind, placebo-controlled, multicentre phase 3 trial. The Lancet Diabetes & Endocrinology.

[cit10] Jastreboff Ania M., le Roux Carel W., Stefanski Adam, Aronne Louis J., Halpern Bruno, Wharton Sean, Wilding John P.H., Perreault Leigh, Zhang Shuyu, Battula Ramakrishna, Bunck Mathijs C., Ahmad Nadia N., Jouravskaya Irina (2024). Tirzepatide for Obesity Treatment and Diabetes Prevention. New England Journal of Medicine.

[cit11] Bliddal Henning, Bays Harold, Czernichow Sébastien, Uddén Hemmingsson Joanna, Hjelmesæth Jøran, Hoffmann Morville Thomas, Koroleva Anna, Skov Neergaard Jesper, Vélez Sánchez Patricia, Wharton Sean, Wizert Alicja, Kristensen Lars E. (2024). Once-Weekly Semaglutide in Persons with Obesity and Knee Osteoarthritis. New England Journal of Medicine.

[cit12] Wilding John P.H., Batterham Rachel L., Calanna Salvatore, Davies Melanie, Van Gaal Luc F., Lingvay Ildiko, McGowan Barbara M., Rosenstock Julio, Tran Marie T.D., Wadden Thomas A., Wharton Sean, Yokote Koutaro, Zeuthen Niels, Kushner Robert F. (2021). Once-Weekly Semaglutide in Adults with Overweight or Obesity. New England Journal of Medicine.

